# Influence of antidepressants on plasma levels of nitric oxide metabolites in patients with major depressive disorder – RETRACTION

**DOI:** 10.1192/bjo.2022.40

**Published:** 2022-03-14

**Authors:** Atsuko Ikenouchi, Naomichi Okamoto, Yusuke Konno, Rintaro Fujii, Yoshihisa Fujino, Reiji Yoshimura

We, the Editors of BJPsych Open, have retracted the following article: Ikenouchi, A., Okamoto, N., Konno, Y., Fujii, R., Fujino, Y., and Yoshimura, R. BJPsych Open (2022). Cambridge University Press 8(1), E14. DOI: https://doi.org/10.1192/bjo.2021.1074

Following publication in December 2021, discrepancies between Table 1 and the Abstract, Results and Discussion sections were discovered and the authors notified. Upon detailed investigation, further statistical and methodological problems were found and the Editors concluded that a corrigendum would not be sufficient to resolve these issues.

The authors of the article agree with the decision.
